# Supplementary material for: Iterative improvement in the automatic modular design of robot swarms
Source: PeerJ Comput Sci. 2020 Dec 7;6:e322. doi: 10.7717/peerj-cs.322 (PMC7924708; doi:10.7717/peerj-cs.322)
Supplement: Supplemental Information 3 [file peerj-cs-06-322-s003.zip › argos3/doc/api/standalone/a00503_source.html]

ARGoS: plugins/robots/generic/control\_interface/ci\_light\_sensor.h Source File


- Main Page
- Related Pages
- Namespaces
- Classes
- Files

- File List
- File Members

# plugins/robots/generic/control\_interface/ci\_light\_sensor.h

Go to the documentation of this file.

```
00001 
00006 #ifndef CCI_LIGHT_SENSOR_H
00007 #define CCI_LIGHT_SENSOR_H
00008 
00009 namespace argos {
00010    class CCI_LightSensor;
00011 }
00012 
00013 #include <argos3/core/control_interface/ci_sensor.h>
00014 
00015 namespace argos {
00016 
00017    class CCI_LightSensor : public CCI_Sensor {
00018 
00019    public:
00020 
00021       virtual ~CCI_LightSensor() {}
00022 
00023       const std::vector<Real>& GetReadings() const;
00024 
00025 #ifdef ARGOS_WITH_LUA
00026       virtual void CreateLuaState(lua_State* pt_lua_state);
00027 
00028       virtual void ReadingsToLuaState(lua_State* pt_lua_state);
00029 #endif
00030 
00031    protected:
00032 
00033       std::vector<Real> m_tReadings;
00034 
00035    };
00036 
00037 }
00038 
00039 #endif
```

---

Generated on 10 Jul 2018 for ARGoS by 
 1.6.1
